# The Tyrosine Kinase c-Src Directly Mediates Growth Factor-Induced Notch-1 and Furin Interaction and Notch-1 Activation in Pancreatic Cancer Cells

**DOI:** 10.1371/journal.pone.0033414

**Published:** 2012-03-30

**Authors:** Yong-Chao Ma, Chong Shi, Yao-Nan Zhang, Lan-Ge Wang, Hao Liu, Hong-Ti Jia, Yu-Xiang Zhang, Fazlul H. Sarkar, Ze-Sheng Wang

**Affiliations:** 1 Department of Biochemistry and Molecular Biology, Cancer Institute, Capital Medical University, Beijing, China; 2 Department of Pathology, Karmanos Cancer Institute, Wayne State University, Detroit, Michigan, United States of America; Hungarian Academy of Sciences, Hungary

## Abstract

The proteolytic activity of Furin responsible for processing full length Notch-1 (p300) plays a critical role in Notch signaling. The amplitude and duration of Notch activity can be regulated at various points in the pathway, but there has been no report regarding regulation of the Notch-1-Furin interaction, despite its importance. In the present study, we found that the Notch-1-Furin interaction is regulated by the non-receptor tyrosine kinase, c-Src. c-Src and Notch-1 are physically associated, and this association is responsible for Notch-1 processing and activation. We also found that growth factor TGF-α, an EGFR ligand, and PDGF-BB, a PDGFR ligand, induce the Notch-1-Furin interaction mediated by c-Src. Our results support three new and provocative conclusions: (1) The association between Notch-1 and Furin is a well-regulated process; (2) Extracellular growth factor signals regulate this interaction, which is mediated by c-Src; (3) There is cross-talk between the plasma growth factor receptor-c-Src and Notch pathways. Co-localization of Notch-1 and c-Src was confirmed in xenograft tumor tissues and in the tissues of pancreatic cancer patients. Our findings have implications for the mechanism by which the Notch and growth factor receptor-c-Src signaling pathways regulate carcinogenesis and cancer cell growth.

## Introduction

Pancreatic cancer has the worst prognosis of all major cancers and remains the fourth most common cause of cancer-related death in the United States and throughout the world [1]. This could be due to the fact that no effective methods of early diagnosis are currently available, as well as the lack of effective therapies. It has been reported that the Notch signaling network is frequently deregulated in human malignancies including pancreatic cancers, with up-regulated expression of Notch receptors and their ligands [2]. Notch signaling is involved in cell proliferation and apoptosis, which affect the development and function of many organs.

*Notch* genes encode proteins that can be activated by interaction with a family of ligands [3]. Notch-1 is present at the cell surface as a heterodimeric molecule (p120/p200), whereas the precursor protein (p300) probably does not reach the cell surface and is cleaved into p120 and p200 in the trans-Golgi network (TGN) by Furin (S1 cleavage) [4], [5]. Ligand binding induces sequential cleavage of Notch receptors, first cleavage of the extracellular domain (ECD) by ADAM (a disintegrin and metalloprotease) proteinase TACE (S2 cleavage) and then of the transmembrane domain by a γ-secretase enzyme complex (S3 cleavage), releasing the intracellular domain (NICD) [3], [6]. This latter then translocates to the nucleus, where it associates with the DNA-binding protein CSL(CBF1/RBPJ-κ) to regulate the transcription of multiple effecter genes, including members of the HES/HEY family [7]. Recently, Lake et al again demonstrated a correlation between loss of cleavage by Furin and loss of *in vivo* function of the Notch receptor, supporting the notion that S1 cleavage is an *in vivo* mechanism controlling Notch-1 signaling [8]. Thus, the proteolytic activity responsible for p300 processing plays a critical role in Notch-1 signaling as it determines the structure of the receptor. However, it is not clear whether cleavage of Notch by Furin is a stochastic, or tightly regulate process.

We screened several kinase inhibitors and found that Src kinase inhibitors inhibited Notch-1 and Furin binding. c-Src is a Mr 60,000 non-receptor tyrosine kinase product of the proto-oncogene c-Src, and the cellular homolog of the Rous sarcoma virus transforming protein, v-Src [10](Ishizawar and Parsons, 2004). Accumulating evidence implicates Src as an important determinant of tumorigenesis, invasion, and metastasis [9]. c-Src is overexpressed in over 70% of pancreatic carcinoma cell lines, and Src kinase activity is often elevated [10]. Thus, Src and Notch-1 are important proteins affecting pancreatic cancer cell growth, invasion and metastasis. In the current study, we detected direct interaction between these proteins. We also found that the interaction between Notch-1 and Furin is not stochastic, but rather well-regulated, since c-Src binds to Notch-1 and stimulates the Notch-1 and Furin interaction. We found that binding of EGFR and PDGFR by their ligands also stimulated the Notch-1-Furin interaction, indicating that extracellular growth factor signals can directly regulate Notch-1 activation in the trans-Golgi apparatus.

## Results

### 1. Effects of Src inhibitors on Furin-induced Notch-1 cleavage

**Figure 1:**
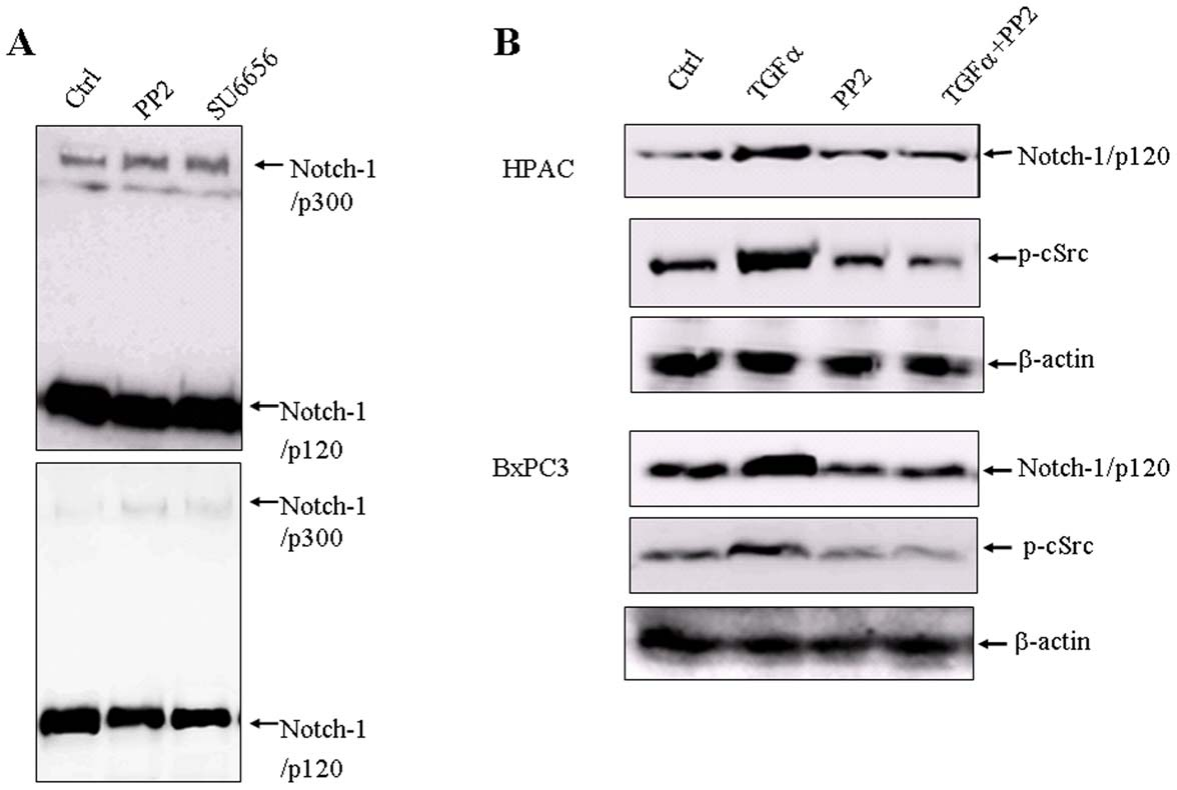
Full-length Notch-1 cleavage is inhibited by Src inhibitors. (A) Src inhibitors PP2 and SU6656 induces inhibition of full-length Notch-1 processing by Furin in HPAC pancreatic cancer cells. HPAC cells were grown in DMEM supplemented with 10% FBS. Cells were treated with 10 µM of PP2 or SU6656 for 60 min. Western blots were performed with anti-Notch-1 antibody. Lower panel, the decrease in Notch-1/p120 was shown in a lighter exposure from a similar Western blot. Notch-1 p300, FL full length Notch proteins; Notch-1 p120, cleaved Notch proteins. (B) The EGFR ligand TGF-α induces full-length Notch-1 cleavage, which is reduced by c-Src inhibitor PP2 in both HPAC and BxPC3 pancreatic cancer cells. Cells were serum-starvated for 48 hours, treated with 10 µM PP2 for 60 min, and then treated with 7 nM TGF-α for 20 min. Western blots were performed with anti-Notch-1, phospho-c-Src, c-Src and β-actin antibodies. p-c-Src, phospho-c-Src.

To investigate which kinase or kinase family is involved in regulation of Furin-induced Notch-1 cleavage, several kinase inhibitors were tested. Proliferating BxPC-3 and HPAC cells were treated with the indicated concentrations of PP2 or SU6656 and the extracts were electrophoresed and blotted for detection of Notch-1. The Src kinase inhibitor PP2 reduced cleavage of full length Notch-1 more than two-fold. After pretreatment with PP2 for 20 min, the 120 kD cleavage products of Notch-1 decreased and full length Notch-1 protein increased (Figure 1A). We also provided a lighter exposure of a similar Western blot in the lower panel of Figure 1A to show the decrease of the 120 kD cleavage product more clearly. PP2-induced inhibition of full length Notch-1 cleavage appeared to be dose-dependent (data not shown).

While it is true that PP2 selectively inhibits Src kinases, at higher concentrations, PP2 is also known to inhibitor EGFR. We conducted dose-response experiments and found that PP2 inhibits c-Src with an IC50 of 4 µM in HPAC cells (Figure S1), but 10 µM PP2 only inhibited EGFR activity by 45%, indicating PP2 inhibits EGFR activity with an IC50 more than 10 µM in the cell culture system (Figure S2). As 10 µM PP2 inhibited c-Src activity by 77%, it seems PP2 inhibits Notch-1 cleavage mainly through c-Src, but not through EGFR, although we could not rule out the possibilty that the direct inhibition of EGFR slightly contribute to PP2-induced down-regulation of Notch-1 cleavage. SU6656, which belongs to a different structural class of Src kinase inhibitors had a similar effect on Notch-1 cleavage as PP2 (Figure 1A).

We also tested whether growth factor-induced c-Src activity affects Notch-1 activation. Proliferating HPAC and BxPC-3 cells were plated on standard growth medium for 24 hours and transferred to serum-free DMEM for 48 hours to permit receptors to equilibrate on the cell surface. They were then treated for 60 minutes with PP2 before stimulation with 7 nmol/L TGF-α. After 20 minutes, whole-cell lysates were prepared, and the extracts were electrophoresed and blotted to detect Notch-1, p-c-Src, and β-actin. Figure 1B demonstrates marked activation of c-Src in the TGF-α-treated cells. Pretreatment of the cells with 10 µM PP2 for 60 min resulted in near complete inhibition of c-Src phosphorylation in all the cell lines tested (Figure 1B). In the HPAC and BxPC3 cells exposed to TGF-α cleavage of Notch-1 increased about 2 and 3-fold, respectively, and these increases were also inhibited by pretreatment with PP2 (Figure 1B).

### 2. TGF-α increases CSL binding to the Hes-1 promoter

**Figure 2:**
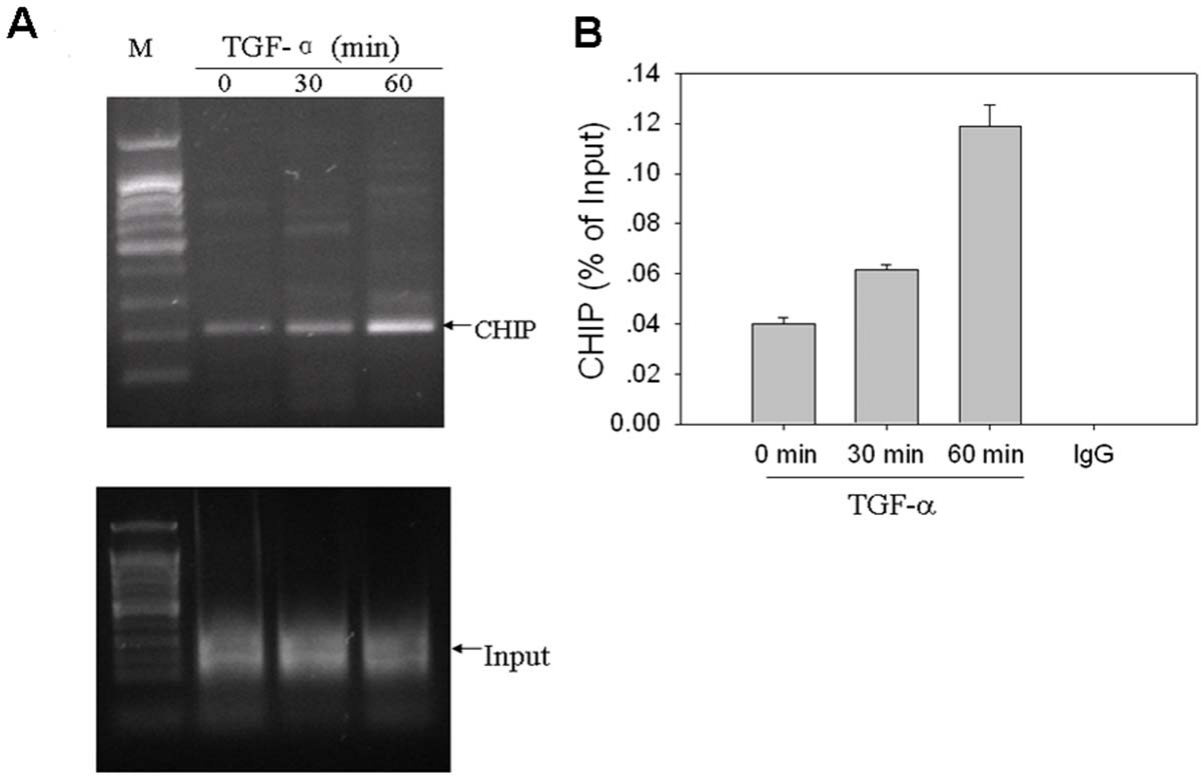
TGF-α induces CSL binding to the Hes-1 promoter. (A) CSL CHIP assay. HPAC cells were grown in serum-free medium for 48 hours, then treated with 7 nM TGF-α for 0, 30, 60 min. Chromatin samples were immunoprecipitated with CSL antibody, DNA was extracted and amplified using Hes-1 promoter primers for 35 cycles by PCR. M, 100 bp DNA ladder. (B) Q-PCR analysis of ChIP DNA samples shows that TGF-α-induced association of CSL-1 with the Hes-1 promoter, resulting in about 0.5, and 2- fold increases by the 30 min and, 60 min time point, respectively. Standard deviations are indicated (n = 3).

After successive cleavage by Furin, TACE and γ-secretase, Notch-1 NICD is released from the plasma membrane and transported to the nucleus where it associates with the DNA-binding protein CSL (CBF1/RBPJ-κ) and induces transcription of multiple effector genes, including Hes-1. To test if TGF-α induces binding of CSL to the Hes-1 promoter we performed CHIP assays. Before TGF-α treatment, a small amount of CSL was detected on the CSL binding site of Hes-1, and this progressively increased following TGF-α treatment for 30 and 60 min (Figure 2A). To better quantify changes in CSL binding to the Hes-1 promoter, we performed Q-PCR assays on the ChIP samples. CSL binding to Hes-1 again increased at 30 min and 60 min after TGF-α treatment (Figure 2B).

We have previously shown that overexpression of NICD increases pancreatic cancer cell proliferation and invasion [11]. In the present study, we have done colonigenic assay and found that overexpression of active form of Notch-1, Notch Extracellular truncation (NEXT), significantly increased HPAC cell colony formation (Figure S3).

### 3. Effects of c-Src inhibitors and growth factors on the Notch-1 and Furin interaction

**Figure 3:**
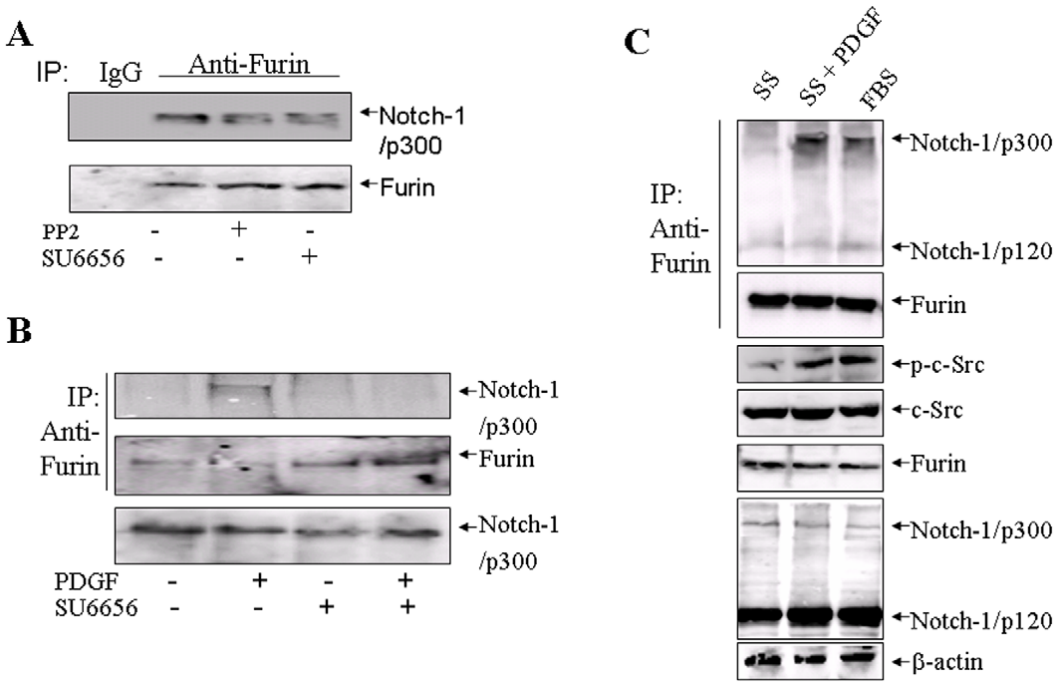
Effects of Src inhibitors PP2 and SU6656 on Notch-1 and Furin association. (A) Src inhibitor PP2 or SU6656 inhibits Notch-1 and Furin association. HPAC cells were grown in DMEM supplemented with 10% FBS without serum starvation. Cells were treated with 10 µM PP2 or SU6656 for 60 min. Normal rabbit IgG was used as a negative control. A densitometry for Figure 3A is provided in Figure S4. (B) PDGF-BB induces the Furin and Notch-1 interaction. Proliferating HPAC cells were serum-starvated for 48 hours, then treated with 20 ng/ml PDGF-BB for 20 min. Normal rabbit IgG was used as a negative control. (C) SU6656 inhibits PDGF-induced Furin-Notch-1 association. Proliferating HPAC cells were serum-starvated for 48 hours, treated with SU6656 for 60 min, and then treated with 20 ng/ml PDGF for 20 min. SS, serum starvation; PDGF, PDGF-BB; FBS, cells were cultured in normal medium, DMEM supplemented with 10% FBS without serum starvation; Notch-1 p120, cleaved Notch proteins; Notch-1 p300, FL Notch proteins. (D) The EGFR ligand TGF-α induces Src activation in Golgi. RFP-B4GLT1-transfected HPAC cells were serum-starvated for 48 h and then treated with 7 nM TGF-α. The cells were stained with anti-phospho-Src (*green*) antibodies to visualize co-localization of phospho-Src (green) and RFP-taged TGN marker B4GALT1. *Bar,* 10 µm.

To determine if c-Src affects Furin activity, we measured the effects of PP2 and SU6656 on Furin activity in BxPC3 and HPAC cells using a fluorescence assay. PP2 and SU6656 only reduced Furin activity by 30% (data not shown), which could not explain 2-fold reduction in Notch-1 cleavage. Therefore we reasoned that c-Src inhibitors might affect the interaction between Notch-1 and Furin. We treated cells with 10 µM PP2 or SU6656 for 20 min and then performed immunoprecipitation assays to look at the association between Furin and Notch-1. PP2 and SU6656 indeed significantly inhibited Furin-Notch-1 binding (Figure 3A). A quantitative densitometry for Figure 3A is provided in Figure S4. At a concentration of 10 µM, PP2 and SU6656 reduced Furin-Notch-1 association by 53% and 43%, respectively (Figure S4).

**Figure 4:**
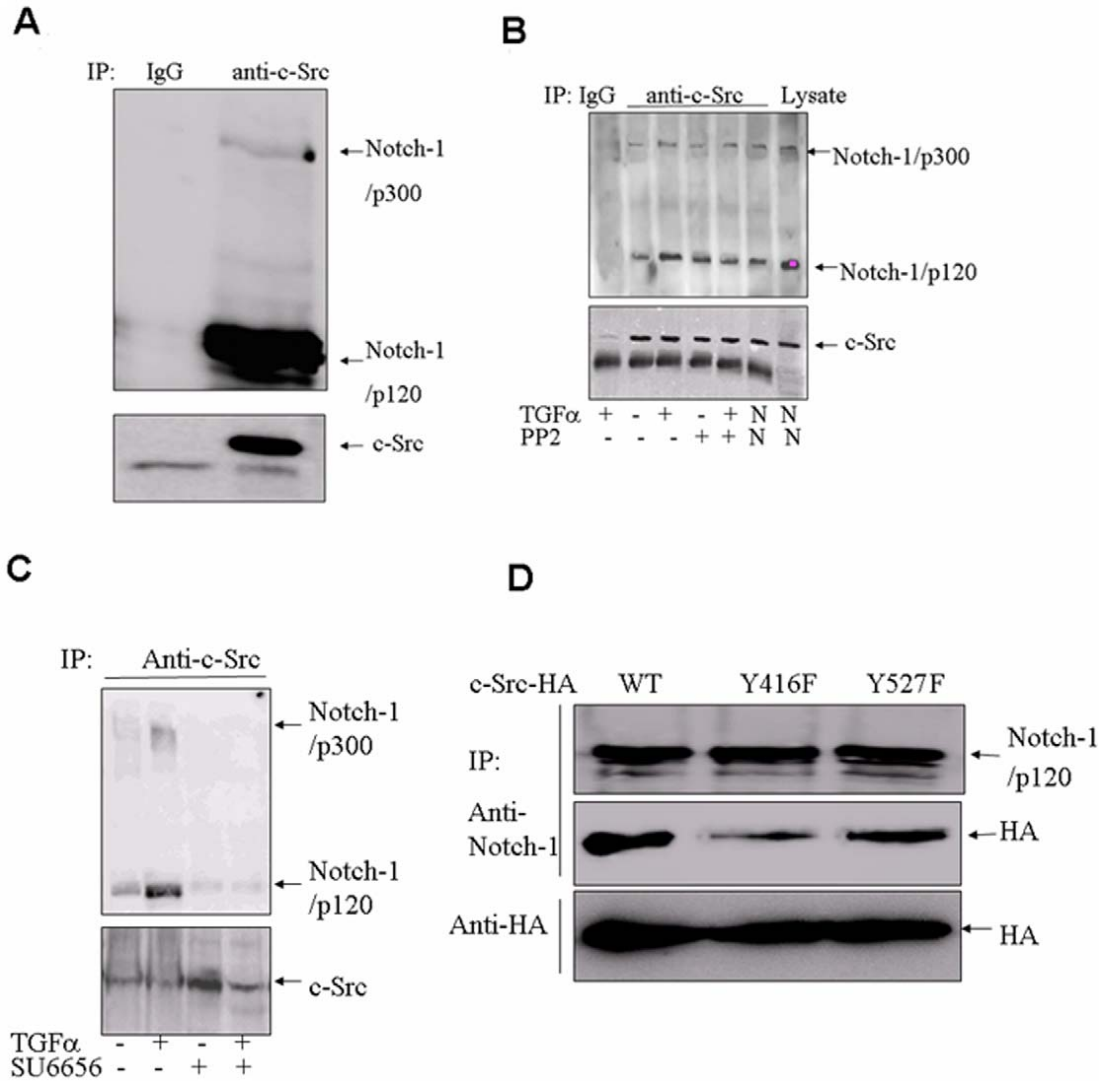
c-Src physically associates with Notch-1 in pancreatic cancer cells. (**A**)** c-Src associatesd with Notch-1 in BxPC3 cells.** Cell lysates were immunoprecipitated with anti-c-Src antibodies, and immunoblotteding (IB) were performed with anti-Notch-1 and anti-c-Src antibodies. Normal rabbit IgGs were used as negative controls. (B) PP2 inhibits TGF-α induced c-Src and Notch-1 association. Serum-starvated HPAC cells were treated with 10 µM PP2 for 60 min, then treated with 7 nmol/L TGF-α for 20 min. Normal rabbit IgGs were used as negative controls for IP. Cells grown in normal medium (DMEM supplemented with 10% fetal bovine serum) without serum starvation were used as positive controls. N, Normal medium. (C) SU6656 inhibits TGF-α-induced c-Src and Notch-1 association. Notch-1/p300., FL Notch proteins; Notch-1/p120, cleaved Notch proteins. (D) Association of c-Src with Notch-1 is dependent on its kinase activity. Wild type, Y416F, and Y527F fused with an HA tag were transiently transfected into HeLa cells. Whole-cell lysates were then prepared, immunoprecipitated with anti-Notch-1 antibodies, and immunoblottedting (IB) were performed with anti-HA and anti-Notch-1 antibodies. Normal rabbit IgG was used as a negative control.

To test whether growth factor-induced Src activity affects Notch-1 activation and the Notch-1-Furin interaction, BxPC-3 and HPAC cells were serum starved for 48 hours and subsequently incubated with 20 ng/ml PDGF-BB for 20 minutes. The results in Figure 3C reveal marked activation of c-Src in the PDGF-BB-treated cells. In these cells co-immunoprecipitation of full-length Notch-1 with Furin increased more than 5-fold (Figure 3B). The main form of Notch-1 associated with Furin was of full-length (Figure 3B), in contrast with the Notch-1 that interacts with c-Src, which was predominantly p120 (Figure 4A). Treatment with 7 nmol/L TGF-α had a similar effect to PDGF-BB (data not shown).

We then tested the effects of c-Src inhibitors on the PDGF-BB-induced increase in Notch-1 and Furin binding. Pretreatment of cells with 10 µM PP2 or SU6656 for 60 min resulted in nearly complete inhibition of the PDGF-BB-induced increase in Notch-1 and Furin binding (Figure 3C). These findings indicate that extracellular growth factor signals mediated by c-Src induce Notch-1 and Furin binding and activate Notch-1.

It is well-accepted that the full length Notch-1 first cleavaged by Furin in the TGN. As growth factor TGF-α and PDGF-BB induces Notch-1-Furin interaction, we asked if growth factor induces c-Src activation in the TGN. We transfected HPAC cells with RFP-taged B4GALT1 as a TGN marker. It was shown that B4GALT1 is mainly located in the TGN, and for some extent, medial Golgi [12]. B4GALT1-transfected HPAC cells were serum-starvated for 48 hours and 7 nM TGF-α was added for 30 min. To determine whether cSrc is activated in the TGN, we looked at co-localization of phospho-Src and B4GALT1 using confocal microscopy. We demonstrated colocalization of RFP-taged B4GALT1 and phospho-c-Src immunoreactivty in TGF-α-treated cells, but not in the control cells (Figure 3D). The results indicates that TGF-α could quickly induces c-Src activation in TGN.

### 4. c-Src directly associates with Notch-1

We asked whether c-Src kinase affects Notch-1 activation directly, or acts indirectly through other effectors. BxPC3 cells were lysed and the lysate was immunoprecipitated with anti-c-Src antibodies, and Notch-1 was detected by Western blotting. Some Notch-1, both full-length Notch-1/p300 and Notch-1/p120, was associated with c-Src (Figure 4A). We also immunoprecipitated Notch-1 and found significant amounts of c-Src in the Notch-1 immunoprecipitates (data not shown). These results indicate that c-Src physically associates with Notch-1.

We then asked if growth factor-stimulated c-Src activation could lead to increased association of c-Src and Notch-1. HPAC cells were serum starved for 48 hours and subsequently incubated with 7 nmol/L TGF-α for 30 minutes. In cells treated with TGF-α, co-immunoprecipitation of both full-length Notch-1 and Notch-1/p120 with c-Src increased about 3 fold, and was inhibited by pretreatment with PP2 (Figure 4B) or SU6656 (Figure 4C).

To demonstrate that Src kinase activity is essential for the association of c-Src with Notch-1, we generated kinase dead and constitutively active src mutants. Kinase activity was disabled by mutating the Y416 tyrosine site to phenylalanine (c-Src-Y416F), while constitutively active Src Y527F was generated by mutating the inhibitory tyrosine 527 to phenylalanine. We overexpressed these mutants in HeLa cells and assessed the ability of the different c-Src constructs to associate with Notch-1. Notch-1 bound to the wild type and constitutive forms of c-Src, but binding to the kinase dead mutant was reduced more than 3-fold (Figure 4D). These results demonstrate that the kinase activity of c-Src is required for efficient binding to Notch-1.

### 5. Co-localization of c-Src with Notch-1 *in vivo*

**Figure 5:**
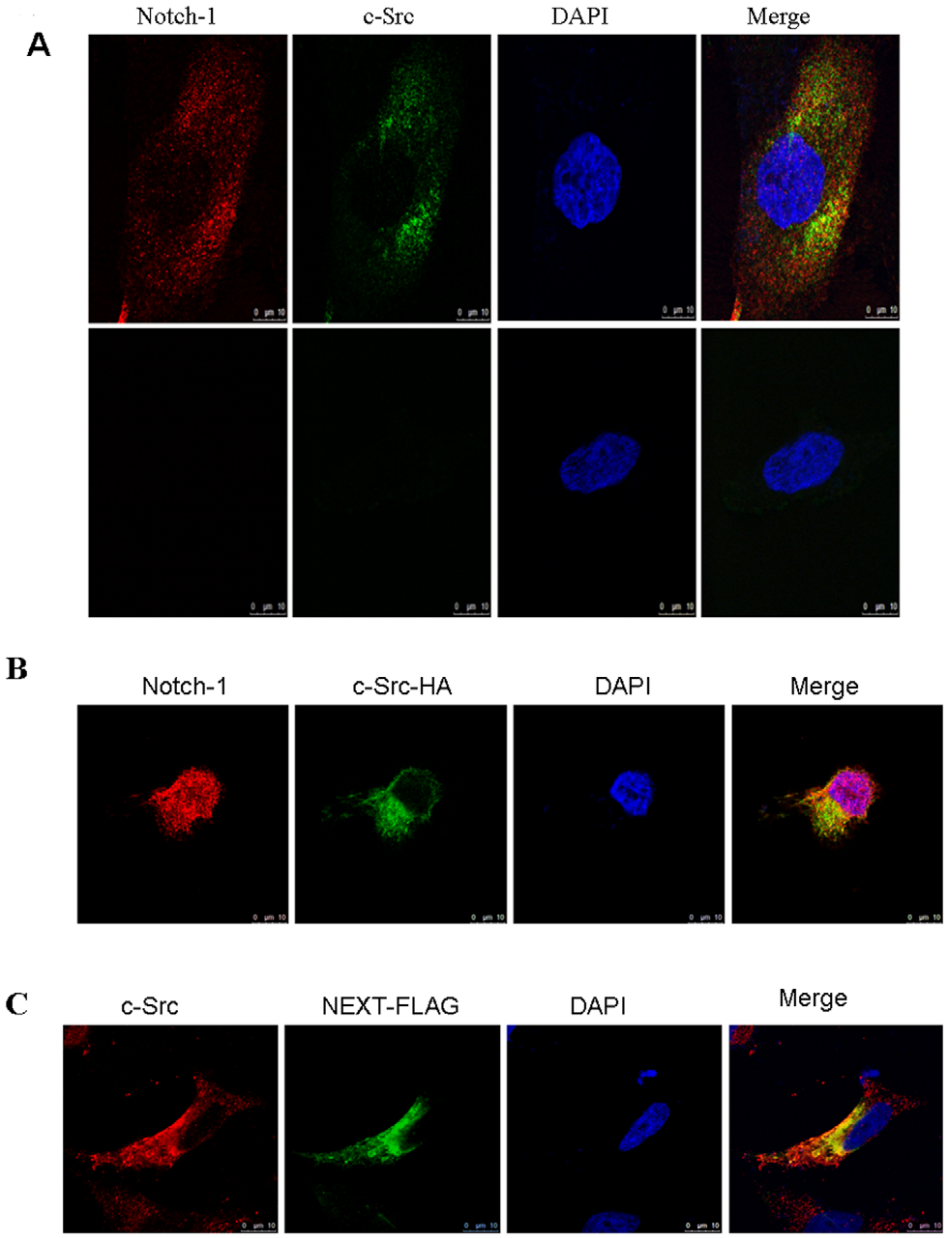
Co-localization and c-Src and Notch-1 *in vivo*. (A) Intracellular interaction of c-Src and Notch1. c-Src and Notch-1 were visualized with a confocal microscope. *Bar,* 10 µm. (B) Co-localization of HA-tagged c-Src and Notch-1. HPAC cell were transfected with pcDNA3-c-Src-HA plasmid, and co-stained with anti-Notch-1 (*red*) and anti-HA (*green*) antibodies to visualize endogenous Notch-1 and HA-tagged c-Src, respectively. *Bar,* 10 µm. (C) Co-localization of endogenous c-Src and FLAG-tagged Notch extracellular truncation (NEXT) fragment. HPAC cell were transfected with pcDNA3-NEXT-FLAG plasmid, co-stained with anti-c-Src (*red*) and anti-FLAG (*green*) antibodies to visualize endogenous c-Src and FLAG-tagged Notch-1 NEXT fragment, respectively. *Bar,* 10 µm.

To determine whether c-Src and Notch-1 co-localize *in vivo*, we performed immunostaining. Colocalization of Notch-1 and c-Src immunoreactivity could be seen from the yellow fluorescence when the images of Cy3-stained anti-Notch-1 and FITC-stained anti-c-Src immunoreactivities were merged (Figure 5A). We then tested for co-localization of HA-tagged c-Src and endogenous Notch-1. Wild-type HA-c-Src was expressed in HeLa cells. Confocal fluorescence microscopy revealed co-localization of c-Src-HA and Notch-1 (Figure 5B), and expression of FLAG-tagged NEXT in HeLa cells revealed co-localization of NEXT-FLAG and c-Src (Figure 5C).

### 6. The protein domains involved in c-Src-Notch-1 binding

**Figure 6:**
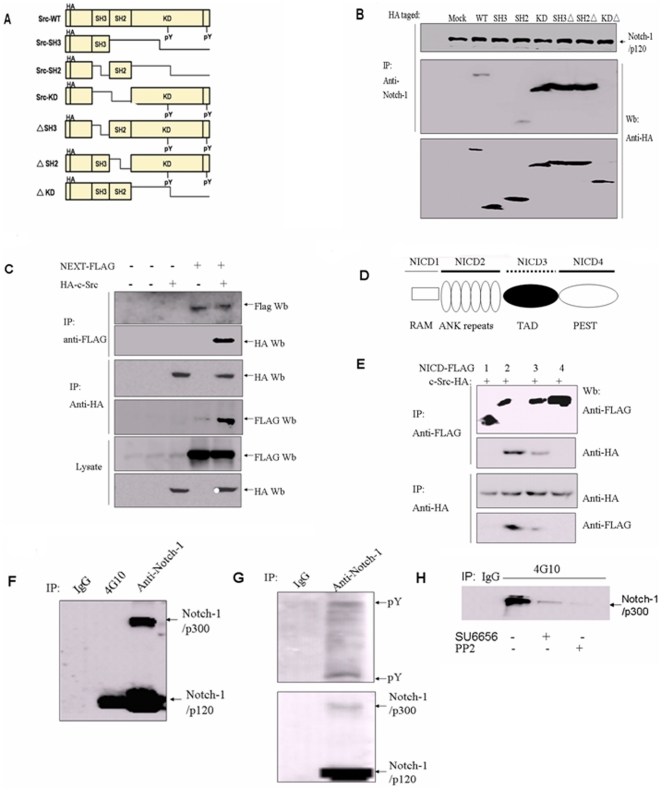
Domain analysis of the c-Src–Notch-1 interaction. The kinase domain of c-Src binds to Notch-1. (A) Schematic representation of the structure of c-Src deletion constructs. (B) The KD domain of c-Src binds to Notch-1 *in vitro*. The indicated fragments of c-Src were fused to an HA tag and transiently transfected into HeLa cells. Whole-cell lysates were then prepared, immunoprecipitated with anti-HA antibodies, and immunoblotteding (IB) were performed with anti-Notch-1 and anti-HA antibodies. Normal rabbit IgG was used as a negative control. (C) Co-immunoprecipitation of FLAG-tagged Notch-1 NEXT and HA-tagged c-Src. (D)Schematic representation of the structure of the Notch-1 deletion constructs. (E) The ankyrin repeat domain of Notch-1 binds to c-Src. The indicated fragments of Notch-1 were fused to a FLAG tag and transiently transfected into HeLa cells. Whole-cell lysates were then prepared, immunoprecipitated with anti-FLAG antibodies, and immunoblotteding (IB) were performed with anti-FLAG and anti-c-Src antibodies. Normal rabbit IgG was used as a negative control. (F) Notch-1 is tyrosine-phosphorylated. Notch-1 was immunoprecipitated with anti-phospho-tyrosine Ab 4G10. Whole-cell lysates of HPAC were prepared, and immunoprecipitated with anti-phospho-tyrosine (4G10), and anti-Notch-1 antibodies, and immunoblotteding (IB) were performed with anti-Notch-1. Normal rabbit IgG was used as a negative control. (G) Detection of tyrosine phosphorylation with anti-Notch-1 immunoprecipitates. Upper panel: Whole-cell lysates of HPAC were immunoprecipitated with anti-IgG and anti-Notch-1 antibodies. The immunoprecipitates were subjected to immunoblotting analysis with anti-phosphotyrosine antibody 4G10. Lower panel, the same blot was stripped, and detected with anti-Notch-1 antibody. (H) Src inhibitor SU6656 or PP2 decreases phospho-tysosine-associated Notch-1. Whole-cell lysates of HPAC were immunoprecipitated with anti-IgG and anti-phosphotyrosine 4G10 antibodies. The immunoprecipitates were subjected to immunoblotting analysis with anti-Notch-1.

c-Src is composed of SH3, SH2 and kinase domains whose ability to bind various proteins have been characterized. We expressed a series of c-Src deletion mutants in HeLa cells to identify the regions necessary for association with Notch-1 (Figure 6A). Association with Notch-1 was reduced if the kinase domain of c-Src was deleted (Figure 6B). In contrast, deletion of the SH3 or SH2 domain did not markedly affect association with Notch-1 (Figure 6B). To determine whether the c-Src KD domain is sufficient for interaction with Notch-1, we performed immunoprecipitation and Western blot assays with a c-Src KD-HA fusion protein in HeLa cell lysates. Notch-1 bound specifically to c-Src KD but not to the c-Src SH3 domain, and only weakly to the SH2 domain (Figure 6B). Thus, the KD domain appears necessary and sufficient for the c-Src interaction with Notch-1.

We also co-transfected FLAG-tagged Notch-1 NEXT and HA-tagged c-Src in HeLa cells. Our results show that NEXT is associated with HA-tagged c-Src (Figure 6C).

Our finding that both full-length Notch-1 (p300) and cleaved Notch-1 (p120) associate with c-Src indicated that the Notch-1 intracellular domain (NICD) has a c-Src binding site. To identify the domain(s) of Notch-1 responsible for c-Src binding, we generated deletion constructs of the NICD (Figure 6D), and performed co-immunoprecipitation analyses with anti-FLAG tag and c-Src-specific antibodies. The results show that the ankyrin repeat (ANK)-deletion mutant had a greatly reduced binding affinity for c-Src, suggesting that the ANK domain of Notch-1 is responsible for binding to c-Src (Figure 6E). Therefore, Notch-1 and c-Src associate via the ANK domain of Notch-1 and the KD domain of c-Src.

To test if Notch-1 is phosphorylated on tyrosine residues, we immunoprecipitated HPAC cell lysates with anti-phospho-tyrosine antibody 4G10, and detected Notch-1 by Western blotting. Some Notch-1/p120 was present in the immunoprecipitate (Figure 6F). We also immunoprecipitated HPAC cell lysates with anti-Notch-1 antibody and detected tyrosine-phophorylation with 4G10 anti-phospho-tyrosine antibody. Both Notch-1/p300 and Notch-1/p120 were phosphorylated on tyrosine (Figure 6G). We treated HPAC cells with 10 µM SU6656 or PP2 for 30 min, and immunoprecipitate the cell lysate with anti-phospho-tyrosine antibody 4G10, then detected phospho-tyrosine-associated Notch-1 (Figure 6H). We found that SU6656 or PP2 markedly decreased phospho-tyrosine-associated Notch-1 (Figure 6H). The results indicated that Notch-1 is potentially phosphorylated by c-Src.

### 7. Co-localization of c-Src and Notch-1 in xenograft pancreatic cancer model

**Figure 7:**
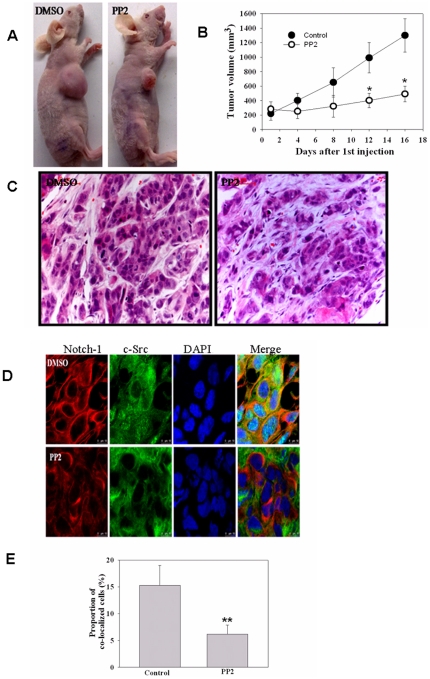
Co-localization of c-Src and Notch-1 in pancreatic cancer xenograft tissues. (A) Effects of PP2 on tumor growth of HPAC pancreatic cancer xenografts in the flanks of nude mice. A representative photograph of one mouse from each group is shown., with tumors located in their flanks. (B) Reduced growth of the xenografts of HPAC pancreatic cancer cells in immunodeficient mice due to PP2 treatment. The tumor volumes were 200–300 mm^3^ at the start of the drugs treatment. Data are presented as mean±SD of tumor volumes. The difference between the DMSO and PP2 groups was highly significant (*P*<0.01; n = 10). (C) Tumors were resected and processed, and the slides were stained with H&E. (D) Immunohistochemical staining in HPAC tumor xenografts. Tumors were resected and processed, and the slides were stained with antibodies to c-Src (red), Notch-1 (green), and DAPI (blue). Localization of c-Src and Notch-1 were visualized by a confocal microscopye. co-localize appear *yellow* in the overlay. *Bar,* 10 µm. (E) The proportion frequency of Red-Green co-localization in the DMSO and PP2-treated tumor tissues (numbers of cells with yellow color/numbers of total cells with blue color in Fig. 7D), was measured with the software Image-Pro Plus software.

To see whether systemic therapy with PP2 could affect the Notch-1-c-Src interaction in xenograft tumors *in vivo*, we established HPAC human pancreatic cancer xenografts in nude mice. Once the HPAC grafts had developed into palpable tumors (200 mg), PP2 was given at 4 mg/kg as s.c. injections for a total of 8 injections every other day. PP2 treatment reduced tumor growth (*P* = 0.0007 versus vehicle) compared with the untreated controls (Figure 7A & B).

Hematoxylin and eosin-stained sections of the xenograft tumors are shown in Figure 7C. Note the nuclear pyknosis and edema seen in the PP2-treated tumor. Immunostaining of serial sections revealed co-localization of c-Src and Notch-1 immunoreactivities in the xenograft tumor tissue (upper panel, Figure 7D), implying a positive correlation between c-Src and Notch-1 activation in individual tumor cells. Treatment with PP2 significantly reduced the co-localization of Notch-1 and c-Src (Figure 7D), the frequency of Red-Green co-localization was much lower than in the control group (6±1.5 versus 15.4±4.6%; *P*<0.01, n = 10; Figure 7E).

## Discussion

Although cleavage of Notch-1 on the S1 site by Furin is necessary for Notch-1 activation, the mechanism governing Notch-1-Furin association was not clear. In the present study we demonstrated that Notch-1 activation is regulated by a tyrosine kinase. We also showed that c-Src is physically associated with Notch-1; c-Src interacts with Notch-1 via its KD domain, and the kinase activity is required for efficient association of c-Src with Notch-1. Extracellular growth factors appear to directly regulate Notch-1 cleavage at the protein level via c-Src.

### Direct cross-talk between growth receptor-c-Src and the Notch pathway

Src family kinases (SFKs) are non receptors kinases overexpressed in the majority of pancreatic cancers and involved in cancer progression and metastasis [9]. Inhibition of these kinases has shown promise in preclinical cancer models. Our results indicate that Notch-1 and c-Src proteins are physically associated and that this association mediates Notch-1 processing and activation. It has been reported that Notch-1 is associated with the serine/threonine kinases GSK3beta and CDK8 [13], [14]. Our results indicates that Notch-1 is also regulated by a tyrosine kinase.

### Notch-1 is potentially a downstream effector of EGFR/PDGFR in pancreatic cancer cells

EGFR signaling impacts many aspects of tumor biology, including proliferation, invasion, spreading, and apoptosis [15]. Activation of EGFR enhances tumor growth, invasion, and spreading; it also inhibits apoptosis. The majority of pancreatic cancers overexpress EGFR, and this has been correlated with advanced disease at presentation and reduced median survival time [16]. EGFR produces its effect on malignant cells via autocrine and paracrine loops and has been shown to bind TGF-α and EGF. EGFR is then autophosphorylated and transphosphorylated on tyrosine residues, resulting in its association with adaptor and signaling molecules and leading to activation of multiple intracellular signaling cascades, including those involving Src, AKT and ras/MAPK1/2 [15].

PDGFR-α and PDGFR-β are structurally similar receptor tyrosine kinases activated by platelet derived growth factor [17]. PDGF induces cell growth, survival [18] and transformation [19]. Activation of PDGFR in tumors can also occur through autocrine or paracrine stimulation as both tumor and normal cells in the stroma secrete PDGF. Over-expression of PDGFR has been found in pancreatic cancers [20]. Like EGFR, PDGFR stimulation leads to activation of intracellular signaling, particularly of Src, AKT, and ras/MAPK1/2.

We have previously observed that over-expression of the Notch-1 intracellular domain (NICD) increases pancreatic cancer cell growth, and knocking down Notch-1 inhibits cell growth [11]. In the present study, we found that overexpression of Notch-1 increases colony formation of HPAC pancreatic cancer cells. We have shown above that activation of either EGFR or PDGFR stimulates Notch-1 activation, which is mediated by c-Src. Thus, Notch-1 activation plays an important role in the growth of pancreatic cancer cells, and in cell proliferation stimulated by activation of EGFR or PDGFR.

In an earlier report we found that down-regulation of Notch-1 decreased cell invasion, whereas Notch-1 overexpression by cDNA transfection led to increased tumor cell invasion [11]. We also found that the down-regulation of Notch-1 reduced NF-κB DNA-binding activity and expression of matrix metalloproteinase-9 (MMP-9) and VEGF [21]. Thus, Notch-1 may act as a down-stream effector of EGFR/PDGFR signaling up-regulating MMP-9 and VEGF expression, and stimulating cell invasion and metastasis. The EGFR/PDGFR-c-Src-Notch may be a key pathway influencing the malignant behavior of tumor cells.

### c-Src KD and Notch-1 ANK domains are involved in the c-Src-Notch-1 interaction

Src family proteins are characterized by four highly conserved Src homology (SH) domains termed SH1 to SH4 [9]. The SH4 domain includes a myristoyl group involved in membrane targeting. SH3 and SH2 domains are protein–protein interaction domains interacting with proline-rich sequences and phosphotyrosine-containing motifs, respectively. A proline-rich linker connects the SH2 domain with the protein kinase KD domain (SH1 domain). Our results show that the KD domain of c-Src is involved in the interaction of c-Src with Notch-1. We also found that human Notch-1 is a tyrosine-phophorylated protein. Regulation of the Notch-1 and Furin interaction depends on Src kinase activity and the KD domain. We are generating mutations of the tyrosine sites in Notch-1 to see which tyrosine residue is required for efficient interaction between Notch-1 and c-Src.

### Plasma membrane signals could reach the TGN and activate Notch-1, and this could be a way to respond to extracellular growth factor stimulation

Previous studies indicate that S1 cleavage of Notch-1 by Furin occurs in the trans-Golgi apparatus and results in the creation of a heterodimeric form of the Notch receptor [4], [5]. This form of the receptor is composed of a 180 kDa cleavage product (NEC) encompassing almost all of the extracellular domain, and a 120 kDa product (NTM) that includes a fragment of the extracellular domain, and the entire transmembrane and intracellular domains. Biotinylation experiments demonstrate that the heterodimeric receptor is the dominant form of Notch located on the cell surface, even though traces of the full-length protein can be detected [8]. Thus, the interaction between Furin and Notch-1 most likely takes place in the TGN.

**Figure 8:**
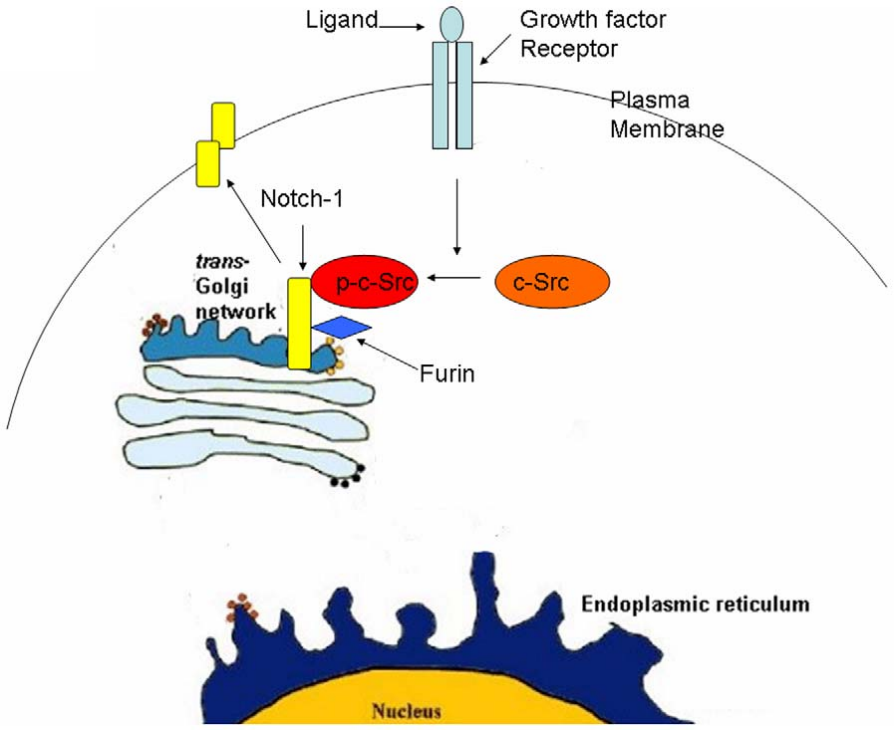
Schematic diagram of c-Src-mediated Notch-1 and Furin interaction. Extracellular growth factor signals induce c-Src activation and stimulate its recruitment to the trans-Golgi network (TGN). Activated c-Src binds to Notch-1 in the TGN, stimulates Notch-1 and furin interaction and Notch-1 cleavage. Notch-1 is present at the cell surface as a heterodimeric molecule and would be able to bind its ligands.

How do the EGFR ligand TGF-α and the PDGFR ligand PDGF-BB induce c-Src activation and Notch-1 cleavage in the TGN? The mechanism underlying growth factor-induced c-Src translocation from the cytosol to the Golgi remains unclear. There are several possibilities: (1) membrane-anchored signaling complexes traffic from the endosomes to the Golgi through Rab9-positive late endosomes [22]; (2) membrane-anchored signaling complexes traffic from endosomes to the Golgi through Rab11-positive recycling endosomes [22]; (3) membrane-anchored signaling complexes traffic from endosomes to the Golgi via AP-1-positive vesicles [23]; (4) A growth factor-induced increase in protein synthesis and cargo loading activates KDEL-R and induces c-Src phosphorylation [24]. A schematic diagram of c-Src-mediated Notch-1 and Furin interaction is provided in Figure 8. The machanisms underlying recruitment of activated c-Src to the TGN deserve further investigation.

Both the Notch-1 and growth factor-c-Src signaling pathways are involved in the progression of tumors in various types of cancer [25], [26]. Therefore, our findings have a potential impact on understanding the mechanism by which Notch-1 and c-Src signaling pathways regulate carcinogenesis and cancer cell growth.

## Materials and Methods

### (1) Cell culture and growth

Human HPAC, BxPX-3 pancreatic cancer cells and HeLa cells (ATCC) were grown in DMEM supplemented with 10% fetal bovine serum (FBS), 100 units/mL penicillin, and 100 µg/mL streptomycin (complete medium) at 37°C in humidified air with 5% CO_2_. Cells were cultured in serum-free medium, and transforming growth factor-α (TGF-α, 7 nmol/L; Invitrogen) or PDGF-BB (20 ng/ml, RnD systems) was added where indicated.

### (2) Expression plasmids

All Notch1-related vectors were based on human sequences. Notch-1 deletions were generated by PCR-based methods with the Notch1-Flag-Myc expression vector as template. Furin-Flag was generated by PCR and cloned into pcDNA3 vector (Invitrogen). HA-tagged c-Src constructs (HA-c-Src) were prepared by amplifying HeLa cDNA with primers 5-CGGGA TCC ATG GGG AGC AGC AAG and 3-G GAA TTCCTATAGGTTCTCTCC. The PCR products were subcloned between the BamHI and EcoRI sites of the mammalian expression vector, pcDNA3-HA. The deletion mutants of Src, SH2-HA, SH3-HA, KD-HA, SH2Δ-HA,SH3Δ-HA, and KDΔ-HA were constructed in pcDNA3-HA by standard PCR cloning. The Tyrosine 416 (kinase-deficient HA-Src Y416F) and 527 Tyr (constitutive active HA-Src-CA Y527F) mutants were created with a QuikChange site-directed mutagenesis kit (Stratagene) with pcDNA3-Src-HA as template. RFP-B4GALT1 plasmid was purchased from Origene.

### (3) Western blotting

Cells were lysed by incubating in RIPA lysis buffer [50 mmol/L Tris (pH 7.5), 100 mmol/L NaCl, 1 mmol/L EDTA, 0.5% NP40, 0.5% Triton X-100, 2.5 mmol/L sodium orthovanadate, 10 µL/mL protease inhibitor cocktail, 1 mmol/L phenylmethylsulfonyl fluoride] for 20 minutes at 4°C. Protein concentrations were determined with the Bio-Rad assay system (Bio-Rad, Hercules, CA). Total proteins were fractionated by SDS-PAGE and transferred onto Immobilon-P transfer membranes (Millipore Corp.). The membranes were blocked for 1 hour with 5% nonfat milk or bovine serum albumin (BSA) in PBS with 0.1% Tween 20. Blots were incubated with primary antibody overnight at 4°C followed by secondary antibodies for 1 hour each at room temperature. Immunoreactive bands were visualized using enhanced chemiluminescence (Pierce). The membranes were then incubated with stripping buffer (Pierce) for 30 minutes at 37°C, re-blocked, and re-probed with β-actin as a loading control.

### (4) Immunoprecipitation and immunoblotting

Cells were washed with cold PBS, collected by scraping, and lysed in RIPA buffer. After standardizing protein concentrations, samples were either prepared for loading on SDS-PAGE or incubated with the appropriate antibody, anti-cSrc (Santa Cruz Biotech), anti-phospho-Src (Cell Signaling), anti-phosphotyrosine (Upstate Biotech), anti-Notch-1 (Santa Cruz Biotech), anti-FLAG (Sigma), anti-HA (Santa Cruz Biotech) or anti-Furin (Alexis) and immunoprecipitated with protein G–Sepharose beads (Amersham-Pharmacia) overnight at 4°C. Immune complexes were denatured by boiling in Laemmli buffer, resolved by SDS-PAGE, and proteins were detected by Western blotting.

### (5) *In vivo* cross-linking and immunoprecipitation (CHIP)

Cell cross-linking was achieved by adding 0.4 ml of 37% formaldehyde (Sigma) to 10 ml of overlaying medium for 10 min at room temperature, followed by the addition of glycine to a final concentration of 125 mM to inactivate the formaldehyde. The chromatin was sheared and cleared by centrifugation (10 min at 17 000 *g*, Eppendorf 5403), split into two 0.5 ml fractions, and used immediately or stored at −70°C. After addition of anti-CSL antibody (Santa Cruz Biotech), 0.5 ml of the sheared chromatin fraction was incubated at 4°C overnight, and transferred to fresh tubes containing 20 µl of washed protein A beads (Amesham-Pharmacia). The slurry was rotated for 45 min (4°C) and the beads were washed five times with 1 ml cold IP buffer without inhibitors. DNA was extracted and used as template in real-time PCR with primers for the promoter of Hes-1 (Sense: 5′TGGCTGAAAGTTACTGTGGG3′, Antisense: 5′TACTGAGCAAGTGCTGAGGG 3′).

### (6) Clonogenic assays

A bottom layer of enriched media plus agar is poured first (2.5 ml), after solidifying this is followed by a layer containing a lower amount of agar and containing 5000 empty vector or pcDNA3-NEXT-transfected cells (cell layer = 5 ml), after solidifying a top layer is poured. The plates are placed in the incubator and after two weeks, colonies are counted by naked eye.

### (7) Confocal microscopy

HPAC cells were plated at a confluence of ∼60% on 12-mm-diameter glass coverslips in six-well plates (Corning, NY). After 24 h, the cells were washed three times with PBS and fixed in 4% formaldehyde for 20 min at room temperature. Following a wash with PBS the cells were blocked with 5% BSA in PBS for 1 h at 37°C and incubated with a 1∶100 dilution of rabbit anti-Notch-1, mouse anti-c-Src (Santa Cruz biotech),Anti-HA (Santa Cruz Biotech), Anti-FLAG (Sigma) overnight at 4°C on a rocking platform. The washed coverslips were incubated for 1 h at room temperature with a 1∶150 dilution of FITC conjugate goat anti-mouse IgG (H+L) for Notch-1, or CY3-conjugated goat anti-rabbit IgG(H+L) (BD Biosciences, USA)(1∶150) secondary antibody for c-Src. The cells were washed again and mounted on glass slides with mounting medium containing4′,6-diamidino-2-phenylindole (DAPI) (Goldenbridge Bio). They were stored in the dark at −20°C until they could be examined with a confocal microscope (LAS AF-TCS SP5, Leica Microsystems). Merging red and green colors yielded yellow, indicating colocalization.

For transfection studies, HeLa cells (5×10^5^/well) were seeded on 12-mm-diameter glass cover slips, placed in 6-well plates and transfected with 0.4 µg of pcDNA3-Flag-NEXT or pcDNA3-c-Src-HA plasmids using Lipofectamine 2000 (Invitrogen), and processed for confocal microscopy.

### (8) Ethics statement

All of the animal experiments were conducted in accordance with the guidelines of Beijing Municipality on the Review of Welfare and Ethics of Laboratory Animals approved by the Beijing Municipality Administration Office of Laboratory Animals (BAOLA). Animal experiments were conducted under the protocol (CCMU-AEC-2010-X-080) approved by the China Capital Medical University Animal Ethics Committee.

### (9) *In Vivo* xenografts

Four-week-old female BALB/cA-nu mice were obtained from the Animal Laboratory of Capital Medical University. Mice were housed in micro-isolator cages with autoclaved bedding in a specific pathogen-free facility with 12-h light/dark cycles. Animals were fed water and food *ad libitum* and were observed for signs of tumor growth, activity, feeding, and pain. Each mouse received 5×10^6^ HPAC cells (in serum-free RPMI 1640) in each flank area. Mice were checked three times a week for tumor development. After palpable tumors (200 mg) had developed, groups of six animals were removed at random and assigned to different groups. Using this model, we studied the effect of PP2. Mice were randomized in two groups: (*a*) treatment group 1, PP2 was given at 4 mg/kg as s.c. injections for a total of 8 injections every other day. (b) the control group received the same volume of 1% DMSO vehicle. Mice in the control and PP2-treated groups were followed to measure s.c. tumors, changes in body weight, and side effects of the drugs. Tumor tissues were harvested for histologic and immunohistochemical analysis.

### (10) Statistics

Control and treated group were compared by Student's t-test. *P* values <0.05 were considered statistically significant.

## Supporting Information

Figure S1 **Dose-response of PP2-induced c-Src inhibition in HPAC cells.** HPAC cells were grown in DMEM supplemented with 10% FBS, and were then treated with various doses of PP2 for 60 min. Western blots were performed with anti-phospho-c-Src (pc-Src) and c-Src antibodies. Lower panel: the histogram shows the quantitative densitometry of phospho-c-Src protein normalized over c-Src expression.
(TIF)

Figure S2 **Dose-response of PP2-induced EGFR inhibition in HPAC cells.** HPAC cells were grown in DMEM supplemented with 10% FBS, and were then treated with various doses of PP2 for 60 min. Western blots were performed with anti-phospho-EGFR (pEGFR) and EGFR antibodies. Lower panel: the histogram shows the quantitative densitometry of phospho-EGFR protein normalized over EGFR expression.
(TIF)

Figure S3 **Clonogenic assay and quantification of HPAC cells after Notch-1 overexpression with NICD cDNA.** (A) representative plates. (B) Colonies were counted. Student's t test was used for statistical analysis. Mock, empty vector-transfected; NICD, Notch intracellular Domain cDNA-transfected; Columns, mean; bars, *SE;* **P<0.01; n = 9.
(TIF)

Figure S4 **Quantitative densitometry for Western blot showed in** **Figure 3A** **.** The histogram shows the quantitative densitometry of Furin-associated Notch-1 protein normalized over Furin protein.
(TIF)
