# Supplementary figures and images for: The Tyrosine Kinase c-Src Directly Mediates Growth Factor-Induced Notch-1 and Furin Interaction and Notch-1 Activation in Pancreatic Cancer Cells
Source: PLoS One. 2012 Mar 30;7(3):e33414. doi: 10.1371/journal.pone.0033414 (PMC3316571; doi:10.1371/journal.pone.0033414)

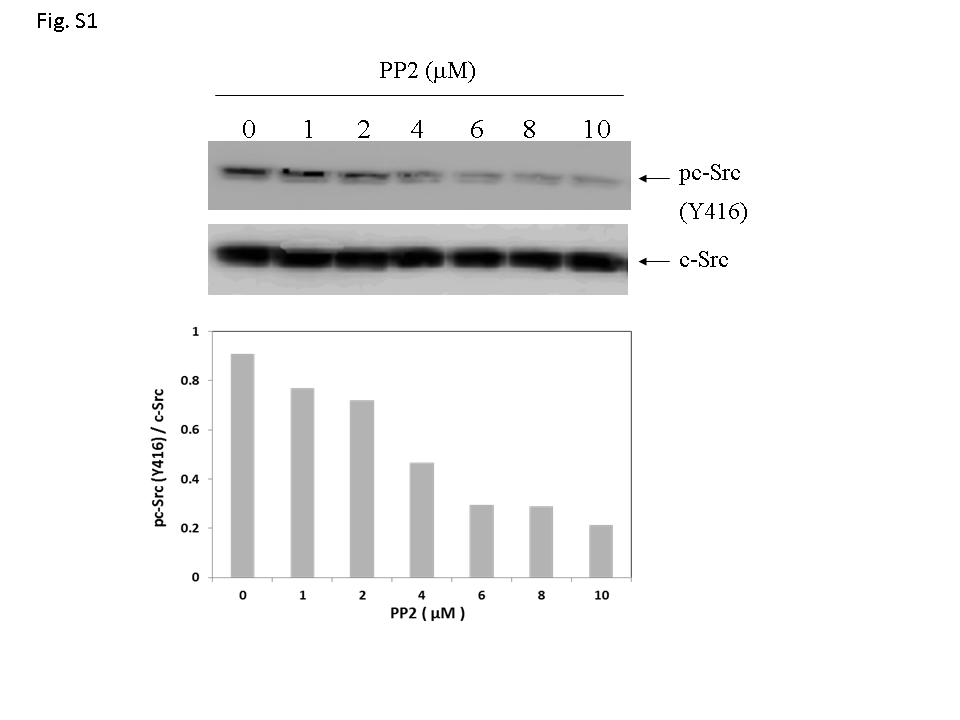

Supplement: Figure S1 — Dose-response of PP2-induced c-Src inhibition in HPAC cells. HPAC cells were grown in DMEM supplemented with 10% FBS, and were then treated with various doses of PP2 for 60 min. Western blots were performed with anti-phospho-c-Src (pc-Src) and c-Src antibodies. Lower panel: the histogram shows the quantitative densitometry of phospho-c-Src protein normalized over c-Src expression. (TIF) [file pone.0033414.s001.tif]

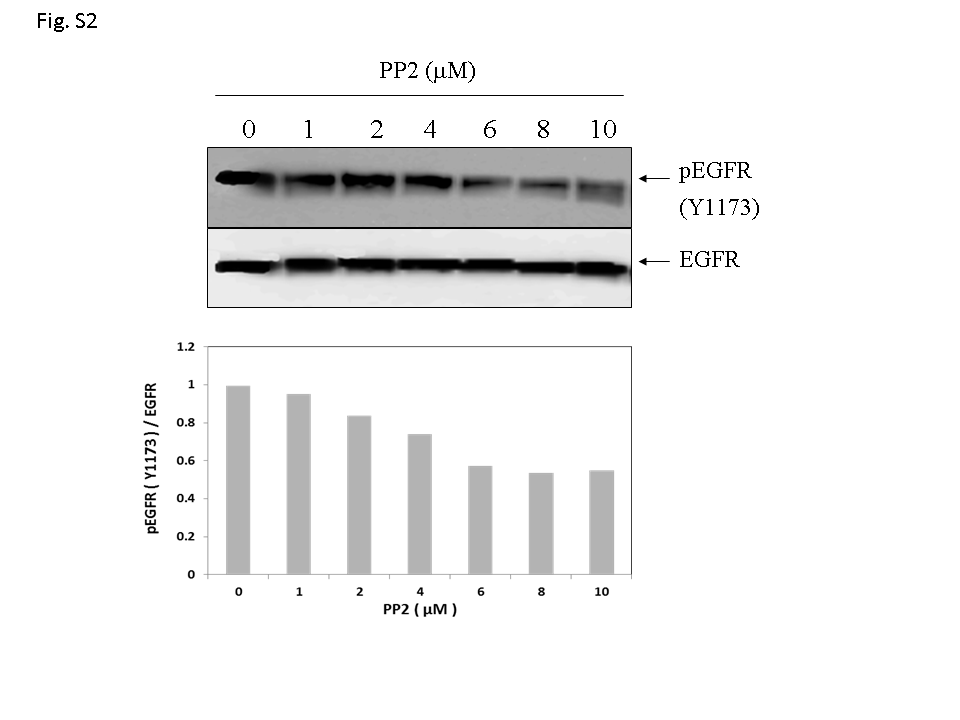

Supplement: Figure S2 — Dose-response of PP2-induced EGFR inhibition in HPAC cells. HPAC cells were grown in DMEM supplemented with 10% FBS, and were then treated with various doses of PP2 for 60 min. Western blots were performed with anti-phospho-EGFR (pEGFR) and EGFR antibodies. Lower panel: the histogram shows the quantitative densitometry of phospho-EGFR protein normalized over EGFR expression. (TIF) [file pone.0033414.s002.tif]

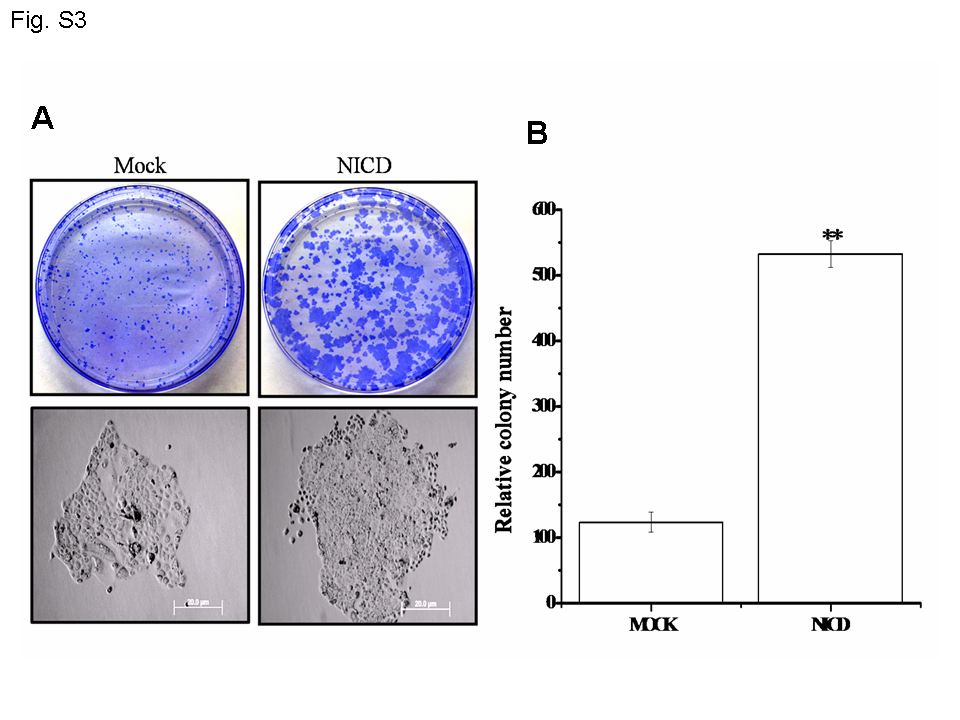

Supplement: Figure S3 — Clonogenic assay and quantification of HPAC cells after Notch-1 overexpression with NICD cDNA. (A) representative plates. (B) Colonies were counted. Student's t test was used for statistical analysis. Mock, empty vector-transfected; NICD, Notch intracellular Domain cDNA-transfected; Columns, mean; bars, SE; **P<0.01; n = 9. (TIF) [file pone.0033414.s003.tif]

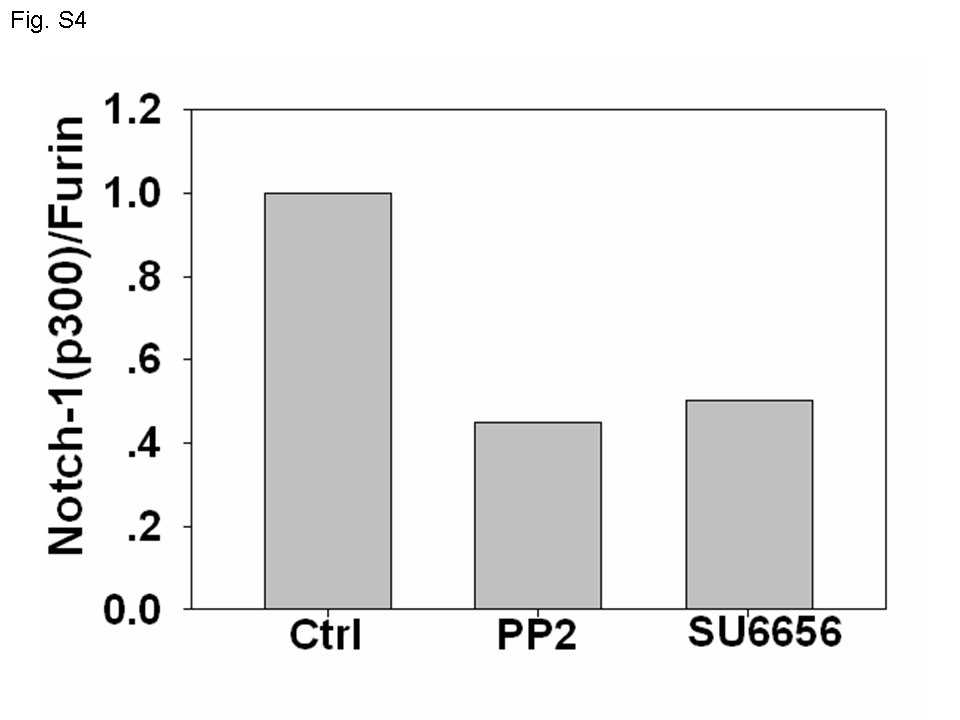

Supplement: Figure S4 — Quantitative densitometry for Western blot showed in Figure 3A . The histogram shows the quantitative densitometry of Furin-associated Notch-1 protein normalized over Furin protein. (TIF) [file pone.0033414.s004.tif]
